# Optimizing Ultraviolet Illumination for Detecting Fluorescent Orthodontic Adhesive Residues during Debonding Procedures

**DOI:** 10.3390/ma17122961

**Published:** 2024-06-17

**Authors:** Grace Chung, Steven Makowka, Stephen Warunek, Thikriat Al-Jewair

**Affiliations:** 1 Private Practice, Brooklyn, NY 11201, USA; gracechu@buffalo.edu; 2 Materials Testing Facility, School of Dental Medicine, University at Buffalo, Buffalo, NY 14214, USA; smakowka@buffalo.edu; 3 Department of Orthodontics, School of Dental Medicine, University at Buffalo, Buffalo, NY 14214, USA; warunek@buffalo.edu

**Keywords:** fluorescence, orthodontics, FIT, UV light, debond, intensity, distance, angulation

## Abstract

Background: Fluorescence-aided identification technique (FIT) studies for orthodontic resins are relatively new, using an arbitrary selection of resins, lights, and work parameters. In order to provide FIT guidelines for optimal visualization, the objectives of this study were to describe the electromagnetic characteristics of fluorescent orthodontic resins, determine appropriate light specification, and describe light and work parameter effects on resin fluorescence. Methods: This in vitro study assessed five fluorescent orthodontic resins and a non-fluorescent control resin using spectrophotometry, a scaled image analysis of 25 μm thick resins to compare intensities, and a visual assessment. Light sources varied by flashlight lens (narrow [N], average [X], and magnified [Z]) and UV intensity (X and X High). Work parameters included distance (20–300 mm) and angulation (15–70°). Visual scores were assigned to determine discernibility. Results: The average excitation maxima was 384 nm. Fluorescence increased with more direct UV light exposure. The highest intensity was recorded with Light X High at 50 mm and 70°. Visual assessment followed image analysis trends, and fluorescence was clinically discernable for all 25 μm thick samples. Conclusions: Excitation wavelength range of 395–405 nm is appropriate for FIT illumination. All resins were anisotropic and showed greater fluorescence with greater angle, higher UV intensity, and closer proximity.

## 1. Introduction

During the orthodontic debond procedure, bulks of adhesive remnants are purposely left after bracket removal since the ideal debond interface is designed to be between the bracket and adhesive layer to minimize enamel damage [1]. This creates a tedious and time-consuming process for the practitioner to remove all adhesive remnants from teeth. With poor resin recognition, adhesive resin may remain, and iatrogenic enamel damage can also occur [1,2,3,4,5,6,7,8]. Thus, accurate identification and removal of adhesive remnants is important to prevent plaque accumulation, caries development, staining, and iatrogenic damage [9,10,11,12].

To aid the detection of adhesives, manufacturers have incorporated fluorescent materials into orthodontic resins, allowing fluorescent resin visualization with ultraviolet (UV) illumination [1,2,5,13,14,15]. Previous studies [1,2,5,6,7,13,15,16] on auxiliary fluorescence-aided identification techniques (FIT) have shown that UV LED lights induce fluorescence of orthodontic resins, improving clinical efficiency and accuracy in removing orthodontic adhesive remnants. However, these studies used arbitrary selection of resins, lighting setups, and working conditions. Consequently, the extent to which these findings can be applied to various clinical scenarios remains unclear, as do the optimal parameters for resin visualization.

Fluorescence intensity is influenced by resin material properties and illumination conditions [17,18,19,20]. Fluorescence occurs when an object absorbs light and emits light of a longer wavelength [17,18,19]. The most effective fluorescence can be obtained when resin is excited by light with a wavelength that induces maximum absorption (maximum excitation wavelength) and is observed at the maximum emission wavelength of the resin [17,18,19]. Currently, there are no spectrophotometry reports on orthodontic resins that provide such electromagnetic information. Therefore, the appropriate FIT light source specification, including ideal excitation wavelength range, remains uncertain. Although reports exist for restorative resins, studies [17,19,20,21,22] show that there is great variability depending on resin brand, shade, and light source UV intensity. Therefore, existing data on restorative resins cannot be readily applied to orthodontic resins. In fact, orthodontic resins exhibited a range of intensities in response to different FIT light sources in clinical studies [6,13,23]. Within the FIT literature for orthodontic resins, different excitation wavelengths have been experimentally used, most commonly ranging from 395 to 410 nm, that produce blue fluorescence [1,2,6,7,13,14,15,16,23,24]. Other wavelengths such as 412 nm [25] or 396 nm [26] induced red fluorescence, and 380–540 nm [27] elicited yellow fluorescence. Alternatively, Tani et al. [28] suggested higher wavelengths such as 430–470 nm to utilize the higher fluorescence of teeth (autofluorescence) to contrast restorative resin. This array of electromagnetic characteristics indicates a need to conduct the spectrophotometry of fluorescent orthodontic resins to determine which excitation wavelength range is appropriate for FIT illumination.

As much as intrinsic electromagnetic properties affect fluorescence, extrinsic factors such as light source specifications, work parameters, and resin thicknesses also elicit variable fluorescence [6,13,20,23,24]. Specifically, our pilot study showed different fluorescence intensities for each orthodontic resin brand with changes in light lens type, wavelength, UV intensity, distance, and angulation. Therefore, a comprehensive consideration must be taken of each resin’s electromagnetic properties, illumination configurations, and light work parameters.

The objectives of this study were to (1) measure maximum and mean excitation and emission maxima (nm) for fluorescent orthodontic resins using spectrophotometry, and (2) determine the effects of light and work variables on fluorescence and clinical discernibility. We hypothesized that fluorescent orthodontic resins would exhibit excitation maxima within the near-UV range. Additionally, fluorescence intensity will increase with greater UV intensity, more perpendicular angles, and closer proximity to the light source.

## 2. Materials and Methods

This two-part in vitro study utilized commercially available orthodontic resins up until 2021 that have fluorescent properties according to manufacturers’ claims. In Part I, the adhesives were identified, and their mean and maximum emission and excitation wavelengths were determined by quantitative fluorescence spectroscopy. In Part II, the optimal light and work parameters for fluorescent resin visualization were determined.

### 2.1. Spectrophotometry

#### 2.1.1. Adhesive Selection

The inclusion criteria for orthodontic resins comprised those indicated by the manufacturer for use as bracket adhesives for direct bonding to enamel, as well as those explicitly stated by the manufacturer to fluoresce or previously demonstrated to fluoresce in the literature. Adhesives that did not show fluorescence in previous studies or no mention of fluorescence by the manufacturer were all excluded.

Five orthodontic resins were included: Opal™ Bond MV (Ultradent Products Inc., South Jordan, UT, USA), BracePaste^®^ (American Orthodontics, Sheboygan, WI, USA), BrackFix^®^ (Voco GmbH, Cuxhaven, Germany), Pad Lock (Reliance Orthodontic Products, Itasca, IL, USA), and GoTo^®^ (Reliance Orthodontic Products, Itasca, IL, USA). As a non-fluorescent resin control, 3M™ Transbond™ XT Light Cure Adhesive (3M Unitek™, St. Paul, MN, USA) was used.

#### 2.1.2. Sample Preparation for Spectrophotometry

**Figure 1:**
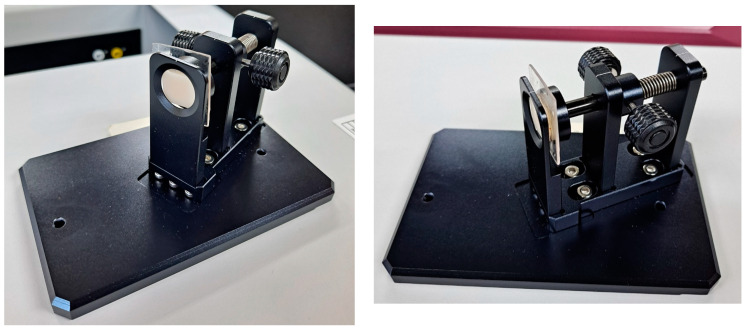
Mounted samples on sample holder for spectrophotometer readings.

Resin samples were prepared in dimension of 4 mm × 15 mm × 2 mm to accommodate for slit size. A thickness of 2 mm was selected to allow adequate fluorescence intensity for detection. Each sample was bonded to a 20 mm × 20 mm × 1 mm rigid slide prepared from clear Invisacryl Ultra^®^ (Great Lakes Orthodontics, Tonawanda, NY, USA) with a plastic conditioner (Reliance Orthodontic Products, Inc., Itasca, IL, USA) and Assure^®^ Plus bonding agent (Reliance Orthodontic Products, Inc., Itasca, IL, USA). The samples were then mounted onto a sample holder for the spectrophotometer (Figure 1).

#### 2.1.3. Spectrophotometry

The maximum excitation and emission wavelengths as well as relative photo-intensities of each resin were recorded using a fluorescence spectrophotometer (Scinco FluoroMate FS-2, Instrument Solutions Benelux BV, Nieuwegein, The Netherlands). The measurements were conducted at a scan speed of 600 nm/min, photo multiplier voltage of 400 V, and slit width of 5 nm. The spectrophotometer recorded data at excitation wavelengths of 350 nm to 500 nm. Emission wavelengths were set to a range from 370 nm to 620 nm.

### 2.2. UV Illumination Parameters

#### 2.2.1. LED Light Selection

Commercially available LED flashlights include 365 and 395 nm, with no other wavelengths in between. Of the two wavelengths, LED flashlights with 395 nm wavelengths were selected since they are closer to the average excitation maxima calculated from Part I. Furthermore, 395 nm was chosen due to UV safety considerations for the operator and the patient. At 395 nm, three commercially available flashlights with narrow (N, 0.50 inch), average (X, 1.18 inch), and magnified/zoomed (Z, 1.50 inch) lens widths were identified. The Z flashlight allows for adjustable magnification, and widening or focusing light. In this study, the focused light was examined. To assess the impact of UV intensity, Light X’s batteries were replaced with higher-powered ones (X High). As a result, Light X High used the same flashlight body as Light X to maintain consistent illumination specifications while only adjusting UV intensity.

#### 2.2.2. Sample Preparation

Lithium disilicate (LDC) blocks (IPS e.max CAD, Ivoclar Vivadent AG, Schaan, Liechtenstein) were prepared using an Isomet^TM^ 1000 Precision Sectioning Saw (Buehler, Lake Bluff, IL, USA) at 300 RPM with 200 g weights. Each B1 shade ceramic block was cut into 10 mm × 15 mm × 3.5 mm rectangular pieces. They were then wet hand-polished for 2 min per abrasive grit, using 300-, 600-, and 1200-grit silicon carbide abrasive paper, respectively. LDC samples were then crystallized following manufacturer’s instructions and using Programat EP 3000 (Ivoclar Vivadent AG, Schaan, Liechtenstein).

**Figure 2:**
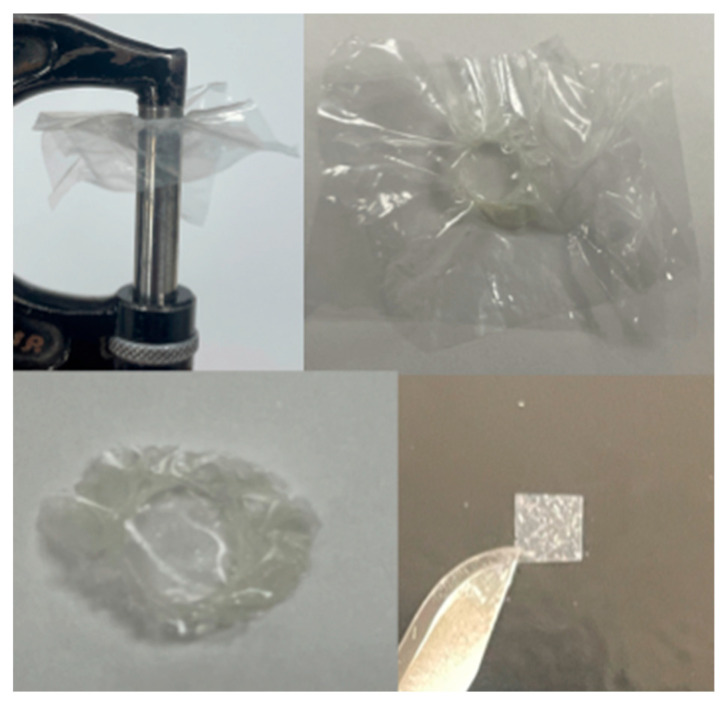
Sample preparation using a micrometer.

Resin samples of 25 μm thickness were then prepared using a micrometer (Figure 2). Each resin sample was sandwiched between two removable filaments of a known thickness, light-cured with a light intensity of 1000 mW/cm^2^ (VALO™, Ultradent Products Inc., South Jordan, UT, USA). The resin samples were excised afterwards into 2.5 mm × 2.5 mm square pieces and re-measured with a micrometer to confirm 25 μm thickness. The resins were then bonded to the center of the LDC using 35% phosphoric acid (Ultra-Etch™, Ultradent Products Inc., South Jordan, UT, USA) and non-fluorescent Assure^®^ Plus Universal Bonding agent.

#### 2.2.3. Flashlight UV Intensity Calibration

Prior to each image capture, a digital UV radiometer (Solarmeter^®^, Model SM 5.0 total UVA+B, Solar Light Company, Inc., Glenside, PA, USA) was used to determine the UV intensity (mW/cm^2^) of each flashlight. The UV intensity was calibrated to 10 mW/cm^2^ at 50 mm for all flashlights (Light N, X, Z) except for Light X High, which was calibrated to 20 mW/cm^2^ at 50 mm.

#### 2.2.4. Digital Image Capture and Conversion

A DinoEye digital microscopy camera (DinoCapture 2.0, Version 1.5.28.D, AnMo Electronics Corporation, Taipei City, Taiwan) was used for image capture using the following fixed parameters: camera distance of 300 mm, shutter speed 1/250 s, aperture value of 13.0, and ISO speed of 1600. Autofocus and automatic white balance were both disabled. Camera, flashlight, and resin sample were fixed onto custom-made mounts to allow for consistent imaging.

Following the conversion protocol of Kim et al. [29] each image was converted to an 8-bit gray scale image, and fluorescence intensity density (a.u.) was calculated using ImageJ Software (version 1.53a, NIH, Bethesda, MD, USA). A 30 × 30 pixel selection was taken at the center of each sample image.

#### 2.2.5. Relative Resin Photointensity According to Work Parameters

Relative fluorescence photointensities (a.u.) of all samples were compared for the three lens types (N, X, Z) and UV intensity (X and X High) according to light angulation and distance. Light angles included 15, 30, 45, 60, and 70° relative to the horizontal plane (90° is perpendicular to the resin surface). Angulations of 75° and higher were not included since the flashlight was brought into camera view, which obstructed resin visualization. For distance, each resin was examined at distances of 20 (approximate handpiece distance), 50, 150, and 300 mm (approximate assistant auxiliary working range).

#### 2.2.6. Visual Assessment

**Figure 3:**
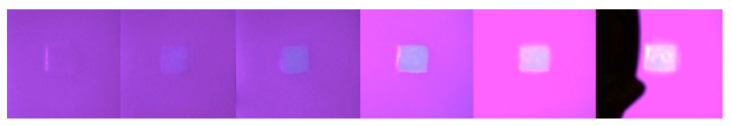
Illuminated resin samples bonded on the center of LDC; from left to right are examples of visual score rubric of 0–5. Note: Far right picture shows the proximity of the flashlight head at 70°. At 75°, the resin view was completely obstructed.

To mimic the operator working distance on a patient, the fluorescence intensity was visually recorded while using orange shield eye protection at a 300 mm distance for all variable combinations. A relative scale of 0–5, adopted and altered from Bush et al. [17] was used, where 0 = no fluorescence detectable, 1 = weak fluorescence, 2 = weak fluorescence with distinct border; 3 = moderate fluorescence; 4 = high fluorescence; and 5 = irradiant fluorescence (Figure 3). A scale of ≤2 was considered clinically indiscernible. All study procedures were completed by the first author.

#### 2.2.7. Statistical Analysis

Images were re-captured after two weeks for all flashlight types at 50 and 150 mm at 45 and 60° to assess intra-examiner reliability. Dahlberg standard deviation was calculated to assess measurement error. The Kolmogorov–Smirnov test and a Q-Q plot were used to examine normality, which were met. A modified population marginal mean was used to account for missing data due to image field obstruction from the flash light head. This obstruction was observed for Light X, X High, and Z at 20 mm for 45, 60, and 70° and light N at 20 mm for 70°. An analysis of variance (ANOVA) was used to measure interactions amongst angulation, distance, and light type followed by a Bonferroni test for pairwise comparisons. Significance level was set at *p* < 0.05, and a confidence interval of 95% was used.

## 3. Results

### 3.1. Spectrophotometry—Excitation Maxima, Emission Maxima, and Fluorescence Intensity

The excitation maxima, emission maxima, and fluorescence intensity for each sample is listed in Table 1. The mean excitation wavelength of fluorescent resins was 384 nm (range = 370–390 nm) and the mean emission maxima was 452 nm (range = 442–465 nm).

### 3.2. Digital Image Analysis and Visual Assessment

#### 3.2.1. Intra-Rater Reliability and Measurement Error

For image capture measurements, the intraclass correlation coefficient value was 0.986, indicating a high reproducibility, and the Dahlberg standard deviation was 3725 a.u.

#### 3.2.2. Digital Image Analysis: Fluorescence Intensity

A comparison of fluorescence intensity between the resin samples is shown in Table 2. The mean fluorescence intensity of Transbond XT was 72,309 (95% CI [71,004, 73,614]), which had no statistical difference from that of LDC (71,310; 95% CI [69,993, 72,627]). Among the florescent resins, the order from highest to lowest mean fluorescence intensity was BrackFix^®^, BracePaste^®^, Opal™ Bond™, Pad Lock, and GoTo^®^.

There was an increase in mean fluorescence intensity with the increase in angle. The mean intensity was highest at 70° (98,987 a.u., 95% CI [98,012, 99,963]). The highest mean fluorescence intensity for distance was at 50 mm (116,972; 95% CI [116,230, 117,714]) and the lowest was at 300 mm (49,934; 95% CI [49,210, 50,657]). However, upon examination of available data at the lower angles—not accounting for modified population mean—values at 20 mm were highest, regardless of angle. For light sources, the order from highest to lowest mean fluorescence was Light X High (98,880; 95% CI [98,096, 99,665]), Light Z (90,891, 95% CI [90,082, 91,699]), Light X (82,138; 95% CI [81,354, 82,923]), and Light N (63,080; 95% CI [62,338, 63,822]).

When the fluorescence intensities were evaluated according to sample, they showed statistically significant differences (F = 125, *p* < 0.001), as shown in Table 3. Similar results were noted when the intensities were evaluated according to angulation (F = 2378, *p* < 0.001), distance (F = 8665, *p* < 0.001), and light type (F = 2331, *p* < 0.001). Additionally, a statistically significant three-way ANOVA interaction between sample, angle, and distance was found (F = 44, *p* < 0.001).

**Figure 4:**
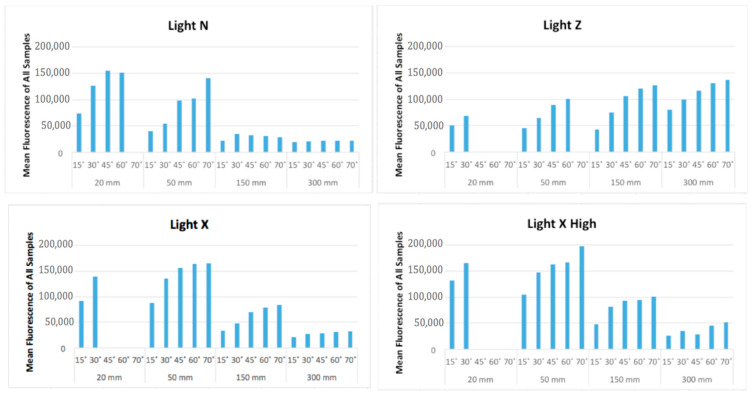
Angle, distance, and fluorescence intensity (a.u.) for flashlights (narrow [N], zoom [Z], average [X], and average with high intensity [X High]).

The post hoc Bonferroni test showed no evidence to reject the null hypothesis that the LDC and Transbond™ XT are different from each other, but they were different from the other five resins. GoTo^®^ was significantly different from all other resins. BracePaste^®^ and BrackFix^®^ were also significantly different from all other resins. Three-way ANOVA for angle, distance, and light mean fluorescence demonstrated that 70° is statistically the best angulation for eliciting the greatest fluorescence (Table 4, Figure 4). It also showed that 20 and 50 mm were the best distances for the highest fluorescence. However, these two distances were not statistically different to each other. For a distance and angulation comparison, the intensity was best at 50 mm and 70°. When comparing distance and light, 50 mm with Light X High was best. For the angulation and light combination, 70° with Light Z had the highest mean intensity. Finally, for the angulation, distance, and light comparison, Light X High at 50 mm and 70° had the highest significant mean fluorescence intensity.

#### 3.2.3. Visual Assessment

Upon visual score analysis, all resins showed fluorescence, except Transbond™ XT, Table 5. The following lists the resins from highest to lowest mean visual score: BrackFix^®^, BracePaste^®^, Pad Lock, Opal™ Bond™ MV, and GoTo^®^.

**Figure 5:**
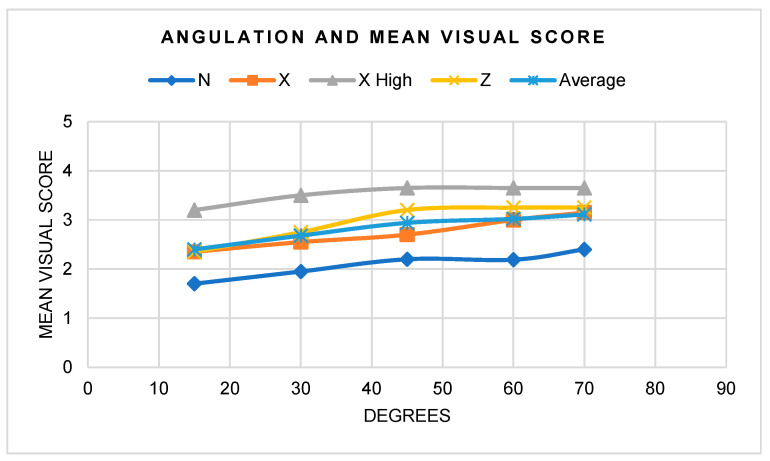
Mean visual score according to angulation.

When the angle was altered, a subtle improvement in scores was noted between increasing angles (Figure 5). Amongst the five angles, an increase in visual score most notably occurred at 45° and 70°. The lowest visual score was predominantly seen at 15°.

**Figure 6:**
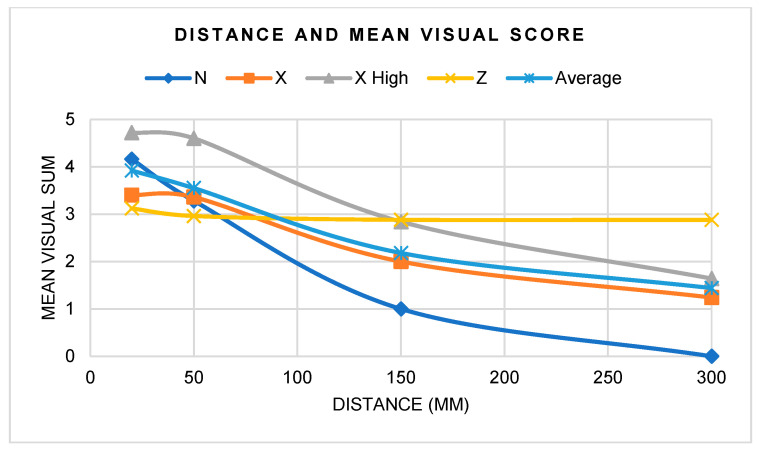
Mean visual score according to distance.

**Figure 7:**
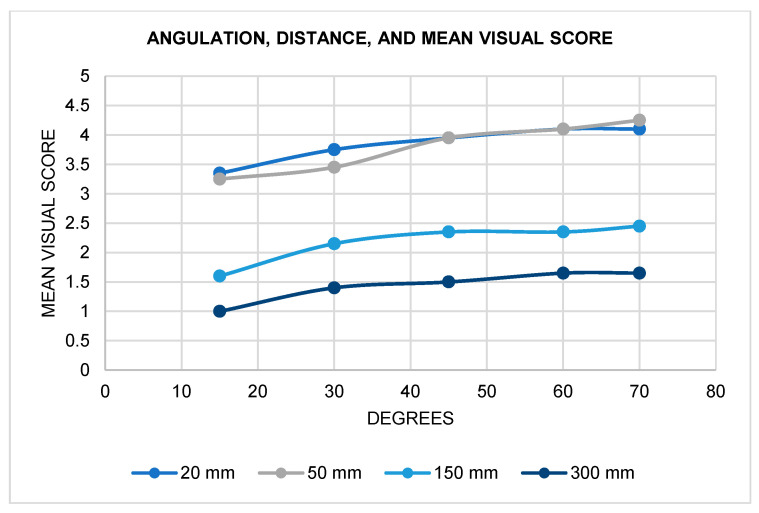
Mean visual score according to angulation and distance.

In regard to distance, fluorescence decreased with increasing distance (Figure 6). When both distance and angle were altered, 20 mm showed clinically acceptable visualization of all samples, at all angles, and for all flashlight types (Figure 7). At 50 mm, the mean score was 3.55, where all samples except GoTo^®^ scored higher than 2. Notably, Light N was not able to elicit any distinguishable fluorescence at 150 and 300 mm, and Light X and X High did not accommodate visibility at 300 mm. At 150 mm, the mean score was 2.18, and Light N had a visual score of 1 for all angles and samples, which is clinically indiscernible for fluorescence visualization. At 300 mm, the mean score was 1.4, with only Light Z having a mean score higher than 2.

## 4. Discussion

This is the first study to investigate the influence of illumination and work parameters on the fluorescence intensity of 25 μm thick resin samples. To date, there are no established FIT clinical guidelines, so clinicians have arbitrarily selected UV light sources and improvised by trial and error to determine useful work parameters for FIT. This study affirmed that near-UV wavelengths, particularly in the range of 395–405 nm, are suitable for FIT light sources for the sample resins. All samples showed different fluorescent intensities, which were dependent on changes in light type and work parameters. Our study also shows for the first time that all sample resins elicit clinically discernable fluorescence at thickness of 25 μm, which makes it the thinnest FIT visible layer reported.

Spectrophotometry showed that all sample resins are fluorescent at near-UV wavelengths, and all resins have different electromagnetic characteristics. Sample resins showed a range of maximum excitation wavelengths from 370 to 390 nm, with an average of 384 nm. This average is lower than the average of 398 nm for restorative resins reported by Meller et al. [19]. The average emission wavelength was 452 nm, which is the same as that of restorative resins [19], demonstrating that resins emit blue fluorescence. All resins had the excitation wavelength bandwidth to be excitable by 395–405 nm lights, confirming that near-UV lights with wavelengths of 395–405 nm are appropriate for eliciting the fluorescence of orthodontic resins. However, since the intensity dropped by an average of 62 (range 55–73%) from 395 to 405 nm, clinicians should note that wavelengths higher than 395 nm are likely to reduce visibility and may differ from the results of this study.

When distinguishing restorative composite resins, Tani et al. [28] recommended using reverse contrast and relying on the higher fluorescence of teeth against resin. For example, they reported that 430, 450, and 470 nm excitation wavelengths elicited high tooth autofluorescence that allowed resin distinction. Our spectrophotometry results confirm nominal fluorescence at 430–470 nm across all resins. To test the autofluorescence contrast method, our pilot study investigated 25 μm samples bonded to a tooth, and they were lit with 395 and 470 nm flashlights (no commercial availability of 430–450 nm flashlights). The 470 nm flashlight showed indistinguishable contrast for all resins against teeth, across all work parameters. In comparison, 395 nm showed distinctly discernable contrast to tooth. The 25 μm resin was most likely too thin for the tooth autofluorescence contrast method; therefore, the study aimed to elicit resin fluorescence.

The current market predominantly offers either 365 or 395 nm for near-UV flashlights, and this study used 395 nm lights. This decision was based on the average excitation maxima of 384 nm being more comparable to 395 nm, safety considerations for the patient and clinician, and the diverse availability of 395 nm flashlights that are feasible for clinical use. Additionally, 395 nm is a safer than 365 nm [28]. Collectively, the absorption spectrum for all sample resins included 395 nm within 15% of peak absorption (highest fluorescence). Therefore, 395 nm is an appropriate wavelength that can elicit optimal fluorescence for all samples.

All samples showed different relative fluorescence intensity. Transbond™ XT was established as a non-fluorescent control since it showed nominal intensity and statistically significant different intensity from that of sample resins. LDC was used to simulate natural tooth visual properties, and spectrophotometry confirmed that LDC has similar electromagnetic wavelength spectra to that of natural teeth; however, fluorescence intensity was nominal. Image analysis showed that amongst the five samples, GoTo^®^ showed the lowest fluorescence and the most difficult visualization. Opal™ Bond™ and Pad Lock showed moderate to high fluorescence. BracePaste^®^ and BrackFix^®^ showed high fluorescence.

Image analysis and perception test demonstrated that all samples are anisotropic, showing mutable fluorescence intensity and visibility with respect to change in light and work parameters. Fluorescence and discernibility increased with more perpendicular angles, decreased with greater distance, and increased with higher UV intensity, thus the null hypothesis was not rejected. From 15 to 70°, the mean intensity of all sample resins increased by 42%, confirming that more perpendicular angles are better for clinical visibility. As for distance, all lights except magnified Light Z showed that fluorescence and visibility decrease with increased distance. Light N, X, and X High were not able to elicit distinguishable visibility at distances greater than 150 mm. Only Light Z was able to elicit visible fluorescence up to 300 mm; interestingly, it showed greater intensity with increased distance. However, this increase in intensity was not visibly remarkable, and visual score showed minimal change amongst all distances. This exception is attributable to the convex magnification lens of Light Z, which causes light to converge to a focal beam, eliciting higher fluorescence. Between the two non-magnified light sources, Light X elicited higher fluorescence compared to Light N, perhaps due to greater number of LED light bulbs of Light X that produced an additive effect. When considering the sole influence of UV intensity between Light X and Light X High, the latter showed 15% greater mean fluorescence intensity and better clinical discernibility. Similarly, our pilot study showed that when batteries with higher energy are used, the lights emit greater UV intensity, and resins could be discerned at distances of 300 mm or greater for all lights. Overall, the highest intensity was recorded at 50 mm and 70° with Light X High. Images at 20 mm were not captured due to image obstruction; however, visually, resins fluoresced brightest at 20 mm. In general, higher fluorescence is noted at closer proximity, more perpendicular angles, and greater UV intensity, most likely due to a more direct exposure to UV light. Overall, this corroborates the finding [20] that UV intensity affects fluorescence intensity. Altogether, clinicians need to take heed of a given resin product and its dependent interplay with light specifications and work parameters, which ultimately affect fluorescence intensity and clinical visibility.

Although other types of lights are available and have been used in other studies, we used flashlights due to their wide variety, clinical feasibility, and applicability for image capture. As for clinical preferences of the type of flashlight, there are different advantages to all three flashlight types. Light N may be favorable for some clinicians due to its low weight, compact body, and pinpoint light surface area. Although Light N did not accommodate discernibility at further distances, visual performance can be enhanced with increased UV intensity using batteries of higher energy. However, precaution must be taken since this method will increase UV exposure for both the patient and operator. Light Z was favorable for consistent visualization for all distances; however, this light was the heaviest and largest of all three lights. Light X covers a fair middle ground of all lights; it is lightweight and has wide light surface area that can visualize multiple intraoral quadrants.

An interesting, novel finding within our study is that all sample resins showed distinguishable fluorescence at thickness of 25 μm, which is the thinnest discernable layer reported to date for FIT visual study. Adhesive remnant heights can be influenced by bracket design and mesh height, and studies have shown varying average adhesive remnant heights, including 0–57 μm [2], 30–100 μm [12], and even up to 229.2 μm [8]. We chose a 25 μm thickness because it was the thinnest reproducible layer with a micrometer, and it simulates adhesive remnants that are more difficult to detect. In literature, there is conflicting information on the minimum detectable thickness of fluorescent resin. One study [24] stated that 50 μm was the minimum thickness that could be visualized with a DSLR and UV camera flash ring at 130 mm. A major limitation of this study [24] is that it appears to include *non-fluorescent* orthodontic resins (ex. Transbond XT) and relied on the contrast of resin to tooth autofluorescence, similar to Tani et al. [28]. Alternatively, Lai et al. [1] stated that FIT left remnants up to 2 μm thick (detection limit is 2 μm). However, instead of the light source truly detecting uniform layer of up to 2 μm, it is more likely that the operator serendipitously removed up to 2 μm, which was likely to be a resin tag. 

Studies have shown variable resin tag lengths, attributable to the viscosity and filler size of resin as well as the etching and bonding method [9,10,30]. A wide range of resin tag lengths are expected, especially depending on resin viscosity and bonding method. For example, low-viscosity resins are shown to have longer resin tags [9]. Zaher et al. [10] have reported orthodontic resin tag lengths of up to 17.4 μm, whereas Kumar et al. [30] reported 53.9 μm resin penetration. In order to disregard resin tag length ambiguity, which may alter sample thickness, it is important to note that our resin layers were prefabricated and then bonded to LDC to ensure 25 μm thickness. Furthermore, to disregard surface irregularity and ensure an even, non-porous surface, 35% phosphoric acid was used instead of hydrofluoric acid. Thus, our samples do not include any resin tags, but the thickness is within the range of resin tag length. Therefore, the visibility of our 25 μm resins suggests a high likelihood of fluorescence visibility of resin tags, and clinicians must be wary of resin tag visibility. Further studies are indicated to evaluate visibility of resin tags in vivo and its clinical implications.

For safety considerations, protective UV eyewear is highly recommended. Although visualization improves with greater UV intensity, a safe and minimal UV intensity range should be used. This recommendation is based on the susceptibility of the patient’s soft tissue to UV exposure. Visible blue-violet light (400–405 nm) may be used to elicit weaker fluorescence; however, one must note that these near-UV visible lights also have the potential to emit UV light (most commercial lights produce a range of excitation wavelengths), and protective eyewear is still recommended.

The findings of our in vitro study must be considered along with their limitations. Fluorescence is affected by multitude of factors, including resin-aging, temperature, bleaching agents, and possibly humidity [31,32,33,34]. This study did not consider these in vivo factors. In one study [34], resins stored in a dark room for 10 years retained 70% fluorescence, so resin aging was not a major concern since orthodontic resins are not subject to such a long-term lifespan. In contrast, Takahashi et al. [35] demonstrated that constant UV light exposure in conjunction with high temperature (63 °C) and 50% humidity reduced resin fluorescence; however, since orthodontic adhesives for fixed appliances are masked under a bracket, they are not subject to constant UV light exposure. The high temperature was suggested to act as a catalyst in reducing fluorescence by photocleaving organic compounds [35]. Our study was conducted at room temperature and without intraoral humidity, so the samples were not subject to any chemical agents that may be subjected in vivo. Since orthodontic resins are not constantly exposed to UV light, higher body temperature was not an immediate concern for expedited resin aging. Furthermore, during debond, resin is subject to room temperature for visualization, and it is also not routine for orthodontic patients to go through bleaching during treatment. For future studies, in vivo temperature and humidity is still recommended since higher temperature and humidity may play a role in fluorescence degradation.

Other limitations included autofluorescence, image capture camera obstruction, and the subjectivity of the visibility test. Although one study [36] showed that the fluorescent properties of LDC are similar to that of natural teeth, the fluorescent intensity of LDC observed in our study was minimal. We nevertheless decided to use LDC instead of teeth because fluorescence differed significantly, even on one surface of a tooth, due to curvature and varying enamel thickness [31]. In our pilot study, the slightest tooth curvature would produce a glare during image capture in respect to alternating light angulation, disrupting calibration. In an attempt to keep uniform background autofluorescence, flat LDC samples were used. Therefore, in vivo clinical discernibility may be different due to tooth autofluorescence, and we recommend future studies on standardized tooth autofluoresecence intensity and its effect on resin discernibility. Furthermore, since the resin layer was prefabricated to ensure uniform thickness, results may vary for different thicknesses and FIT methods of alternate surface, etch, and bonding agents. During image capture, there were also physical limitations. Measurements beyond 70° were not feasible due to the obstruction of camera view from the flashlight head. Image capture was also similarly obstructed for Light Z at 20 and 50 mm. Moreover, the visibility test is subjective, so discernibility will vary amongst clinicians.

Overall, our study provides insight on the trends of how fluorescence intensity alters in respect to the resin, light source, and work parameter. As there are continuous modifications to resin manufacture and light specification, fluorescence discernment will also change. Discernment will also vary greatly depending on the clinician’s perception and proficiency. Therefore, clinicians must be cognizant on how fluorescence intensity alters depending on the aforementioned variables, determine the appropriate level of fluorescence intensity they are comfortable with, and adjust FIT work variables accordingly.

## 5. Conclusions

This study concluded the following:

- The appropriate wavelengths for FIT light specification are 395–405 nm; wavelengths closer to 395 nm allow for better visualization.
- All resin samples showed different relative fluorescence intensity and anisotropic fluorescence at 25 μm, except for the Transbond XT control.
- There were statistically significant differences between distances, angles, and light sources, as well as interactions when considered as pairs; fluorescence intensity increased with more perpendicular angles and with greater UV intensity, but decreased with increased distance.
- The highest fluorescence was recorded with a combination of Flashlight X High at 50 mm and 70°.

## Figures and Tables

**Table 1:** Spectrophotometry readings.

Sample	Excitation Maxima (nm)	Emission Maxima (nm)
Lithium Disilicate	350	545
Transbond™ XT	370	405
GoTo^®^	390	442
BracePaste^®^	370	446
Opal™ Bond™ MV	380	465
Pad Lock	390	460
BrackFix^®^	390	446
Mean of fluorescent resins	384	452

**Table 2:** Mean fluorescence intensity by resin type, working angle, distance, and light type.

			95% Confidence Interval	
Variable		Mean (a.u.)	Lower Bound	Upper Bound
**Resin**	Transbond™ XT	72,309.5 ^a^	71,124.8	73,494.3
	GoTo^®^	75,379.2 ^a^	74,194.5	76,564.0
	BracePaste^®^	85,796.9 ^a^	84,612.2	86,981.7
	Opal™ Bond™ MV	83,415.8 ^a^	82,231.1	84,600.6
	Pad Lock	82,068.8 ^a^	80,884.0	83,253.6
	BrackFix^®^	88,564.9 ^a^	87,380.1	89,749.6
**Angle**	15°	57,529.4	56,720.8	58,338.0
	30°	82,760.3	81,951.7	83,569.0
	45°	89,041.6 ^a^	88,144.5	89,938.7
	60°	95,313.2 ^a^	94,416.1	96,210.3
	70°	98,987.8 ^a^	98,012.6	99,963.1
**Distance**	20 mm	115,470.6 ^a^	114,447.8	116,493.5
	50 mm	116,972.9 ^a^	116,230.8	117,714.9
	150 mm	67,712.1	66,988.8	68,435.4
	300 mm	49,934.1	49,210.8	50,657.4
**Light**	Light X	82,138.5 ^a^	81,354.0	82,923.0
	Light Z	90,891.3 ^a^	90,082.6	91,699.9
	Light N	63,080.5 ^a^	62,338.4	63,822.5
	Light X High	98,880.6 ^a^	98,096.1	99,665.1

^a^ Based on modified population marginal mean.

**Table 3:** Tests of between-subjects effects.

Source	df	Mean Square	F	Sig. *
S (Sample)	4	1,688,718,239.1	125.1	0.000
A (Angle)	4	32,105,679,385.7	2378.7	0.000
D (Distance)	3	116,964,668,292.3	8666.0	0.000
Light type	3	31,467,684,346.6	2331.5	0.000
D * A	11	2,901,121,459.6	214.9	0.000
A * Light	12	824,333,791.7	61.1	0.000
D * Light	9	19,285,101,263.8	1428.8	0.000
D * A * Light	26	604,203,700.0	44.8	0.000

* ANOVA test, *p* < 0.05 was considered statistically significant; R^2^ = 0.991 (Adjusted R Squared = 0.989).

**Table 4:** Three-way ANOVA for angle, distance and light mean fluorescence (a.u.).

Angle	Distance	Light Type	Mean	95% Confidence Interval	
				Lower Bound	Upper Bound
15°	20 mm	Light X	92,219.0	88,984.4	95,453.6
		Light Z	50,971.0	47,736.4	54,205.6
		Light N	73,483.0	70,248.4	76,717.6
		Light X High	132,090.6	128,856.0	135,325.2
	50 mm	Light X	87,551.2	84,316.6	90,785.8
		Light Z	45,390.8	42,156.2	48,625.4
		Light N	39,913.6	36,679.0	43,148.2
		Light X High	104,626.8	101,392.2	107,861.4
	150 mm	Light X	33,586.6	30,352.0	36,821.2
		Light Z	42,913.6	39,679.0	46,148.2
		Light N	22,211.4	18,976.8	25,446.0
		Light X High	48,250.4	45,015.8	51,485.0
	300 mm	Light X	20,721.6	17,487.0	23,956.2
		Light Z	79,919.6	76,685.0	83,154.2
		Light N	20,073.8	16,839.2	23,308.4
		Light X High	26,547.4	23,312.8	29,782.0
30°	20 mm	Light X	139,171.8	135,937.2	142,406.4
		Light Z	68,022.8	64,788.2	71,257.4
		Light N	126,755.0	123,520.4	129,989.6
		Light X High	165,535.6	162,301.0	168,770.2
	50 mm	Light X	135,170.0	131,935.4	138,404.6
		Light Z	64,459.2	61,224.6	67,693.8
		Light N	54,583.6	51,349.0	57,818.2
		Light X High	147,866.4	144,631.8	151,101.0
	150 mm	Light X	48,188.2	44,953.6	51,422.8
		Light Z	75,228.8	71,994.2	78,463.4
		Light N	35,644.8	32,410.2	38,879.4
		Light X High	81,123.6	77,889.0	84,358.2
	300 mm	Light X	27,274.6	24,040.0	30,509.2
		Light Z	99,632.4	96,397.8	102,867.0
		Light N	20,865.4	17,630.8	24,100.0
		Light X High	34,643.0	31,408.4	37,877.6
45°	20 mm	Light X	. ^a^	.	.
		Light Z	. ^a^	.	.
		Light N	154,800.8	151,566.2	158,035.4
		Light X High	. ^a^	.	.
	50 mm	Light X	156,086.4	152,851.8	159,321.0
		Light Z	88,884.2	85,649.6	92,118.8
		Light N	97,757.4	94,522.8	100,992.0
		Light X High	162,382.6	159,148.0	165,617.2
	150 mm	Light X	69,465.6	66,231.0	72,700.2
		Light Z	106,261.6	103,027.0	109,496.2
		Light N	33,041.0	29,806.4	36,275.6
		Light X High	93,562.0	90,327.4	96,796.6
	300 mm	Light X	28,220.8	24,986.2	31,455.4
		Light Z	116,845.6	113,611.0	120,080.2
		Light N	22,201.4	18,966.8	25,436.0
		Light X High	28,031.8	24,797.2	31,266.4
60°	20 mm	Light X	. ^a^	.	.
		Light Z	. ^a^	.	.
		Light N	151,656.8	148,422.2	15,4891.4
		Light X High	. ^a^	.	.
	50 mm	Light X	164,408.2	161,173.6	167,642.8
		Light Z	101,201.2	97,966.6	104,435.8
		Light N	101,890.4	98,655.8	105,125.0
		Light X High	166,721.2	163,486.6	169,955.8
	150 mm	Light X	79,018.2	75,783.6	82,252.8
		Light Z	119,993.2	116,758.6	123,227.8
		Light N	30,985.6	27,751.0	34,220.2
		Light X High	93,858.8	90,624.2	97,093.4
	300 mm	Light X	31,758.6	28,524.0	34,993.2
		Light Z	130,085.4	126,850.8	133,320.0
		Light N	21,760.2	18,525.6	24,994.8
		Light X High	45,733.6	42,499.0	48,968.2
70°	20 mm	Light X	. ^a^	.	.
		Light Z	. ^a^	.	.
		Light N	.^a^	.	.
		Light X High	. ^a^	.	.
	50 mm	Light X	166,051.6	162,817.0	169,286.2
		Light Z	. ^a^	.	.
		Light N	140,271.2	137,036.6	143,505.8
		Light X High	197,268.2	194,033.6	200,502.8
	150 mm	Light X	84,667.6	81,433.0	87,902.2
		Light Z	126,852.4	123,617.8	130,087.0
		Light N	28,986.2	25,751.6	32,220.8
		Light X High	100,402.4	97,167.8	103,637.0
	300 mm	Light X	32,795.0	29,560.4	36,029.6
		Light Z	137,598.4	134,363.8	140,833.0
		Light N	21,647.6	18,413.0	24,882.2
		Light X High	52,325.6	49,091.0	55,560.2

^a^ This level combination of factors is not observed; thus, the corresponding population marginal mean was not estimable.

**Table 5:** Flashlight mean visual score according to distance.

	N	X	X High	Z	Average
20 mm	4.2	3.4	4.7	3.1	3.9
50 mm	3.3	3.4	4.6	3.0	3.6
150 mm	1.0	2.0	2.8	2.9	2.2
300 mm	0.0	1.2	1.6	2.9	1.4
**Average**	2.1	2.5	3.5	3.0	

## Data Availability

No new data were created or analyzed in this study.
